# Curative effects of traditional Chinese medicine on liver fibrosis: A protocol for a systematic review and meta-analysis

**DOI:** 10.1097/MD.0000000000024587

**Published:** 2021-03-05

**Authors:** Xia Gao, Wenfu Cao

**Affiliations:** College of Traditional Chinese Medicine, Chongqing Medical University, Chongqing, China.

**Keywords:** liver fibrosis, systematic review, traditional Chinese medicine

## Abstract

**Background:**

Assessing the effectiveness and safety of traditional Chinese medicine on liver fibrosis is the main purpose of this systematic review protocol.

**Methods:**

The following electronic databases will be searched from their respective inception dates to 1st December 2021: PubMed, MEDLINE, the Cochrane Library, Embase, WorldSciNet, Ovid, the Allied and Complementary Medicine Database, the Web of Science, Chinese Biomedical Literature Database, China National Knowledge Infrastructure, the Chongqing VIP Chinese Science and Technology Periodical Database, the Wanfang Database, and the China Biology Medicine Disc. All published randomized controlled trials in English or Chinese related to curative effects of Traditional Chinese medicine on liver fibrosis will be included. The primary outcome is the levels of serum hyaluronic acid, laminin, type III procollagen, and type IV procollagen. There is no secondary outcomes. Two reviewers will conduct the study selection, data extraction, and assessment independently. The assessment of risk of bias and data synthesis will be conducted with Review Manager Software V.5.2.

**Results:**

The results will provide a high-quality synthesis of current evidence for researchers in this subject area.

**Conclusion:**

The conclusion of our study will provide an evidence to judge whether traditional Chinese medicine is an effective intervention for patients with liver fibrosis.

**Registration number:**

INPLASY202110017

## 1 Introduction

Chronic liver disease will contribute to liver fibrosis, which features replacement of liver tissue by fibrosis, scar tissue, and regenerative nodules, leading to liver dysfunction.^[1]^ This will lead to impairment of liver and affect patients’ quality of life.^[2]^ Thus, the prevention and treatment of liver fibrosis is significant. Nowadays, there is no specific treatment, despite of early intervention or control of etiologies and hepatic inflammation and regulation of hepatic extracellular matrix metabolism and stellate cell activation.^[3]^

It has been reported that traditional Chinese medicine could be effective to the liver fibrosis.^[4]^ This review is to judge whether traditional Chinese medicine is an effective intervention for patients with liver fibrosis.

## 2 Methods and analysis

### 2.1 Study registration

This systematic review protocol was registered with PROSPERO 2020 (registration number: INPLASY202110017). And the protocol report is in the base of the preferred reporting items for systematic reviews and meta-analyses protocols (PRISMA-P) declaration guidelines.^[5]^ The review will be performed in line with the PRISMA-P declaration guidelines.^[6]^

### 2.2 Inclusion criteria for study selection

#### 2.2.1 Type of study

All randomized controlled trials (RCTs) about traditional Chinese medicine on liver fibrosis which were reported in English and Chinese will be included. Trials with 2-arm or 3-arm parallel design will be also included. Non-RCTs, quasi-RCTs, case series, reviews, animal studies, and any study with a sample size of less than 10 participants will be excluded.

#### 2.2.2 Type of participant

Participants who were 18 years or older with liver fibrosis will be included in spite of the gender, race, education, or economic status.

#### 2.2.3 Type of intervention

Experimental interventions include traditional Chinese medicine therapy. Control interventions would be western medicine therapy.

#### 2.2.4 Type of outcome measure

The primary outcome will be the levels of serum hyaluronic acid, laminin, type III procollagen, and type IV procollagen. There are no secondary outcomes.

### 2.3 Search methods for identification of studies

#### 2.3.1 Electronic data sources

The following electronic databases will be searched from their respective inception dates to 1st December 2021: PubMed, MEDLINE, the Cochrane Library, Embase, WorldSciNet, Ovid, the Allied and Complementary Medicine Database, the Web of Science, Chinese Biomedical Literature Database, China National Knowledge Infrastructure, the Chongqing VIP Chinese Science and Technology Periodical Database, the Wanfang Database, and the China Biology Medicine Disc. All published randomized controlled trials in English or Chinese related to traditional Chinese medicine on liver fibrosis will be included.

#### 2.3.2 Searching other resources

The reference lists of potentially missing eligible studies will be scanned ant the relevant conference proceedings will be scanned as well.

#### 2.3.3 Search strategy

The search strategy for PubMed is shown in Table 1. The following search keywords will be used: Traditional Chinese medicine (eg, “Chinese Drugs, Plant” or “Chinese Herbal Drugs” or “Herbal Drugs, Chinese” or “Plant Extracts, Chinese” or “Chinese Plant Extracts” or “Extracts, Chinese Plant ”); Liver Fibrosis. (eg, “Liver Cirrhosis” or “Liver Cirrhosis, Biliary” or “Liver Cirrhosis, Alcoholic”); randomized controlled trial (eg, “randomized controlled trial” or “controlled clinical trial” or “random allocation” or “randomized” or “randomly” or “double-blind method” or “single-blind method” or “clinical trial”. The equivalent search keywords will be used in the Chinese databases.

**Table 1:** Search strategy for the PubMed database.

Number	Search items
1	Liver Fibrosis. Mesh.
2	Liver Fibrosis. ti, ab
3	1 or 2
4	Liver Cirrhosis. Mesh
5	Liver Cirrhosis. ti, ab
6	4 or 5
7	Liver Cirrhosis, Biliary. Mesh
8	Liver Cirrhosis, Biliary. ti, ab
9	7 or 8
10	Liver Cirrhosis, Alcoholic. Mesh
11	Liver Cirrhosis, Alcoholic. ti, ab
12	10 or 11
13	Medicine, Chinese Traditional. Mesh
14	Traditional Chinese medicine.ti, ab
15	Chinese medicine. ti, ab
16	Chines herbs. ti, ab
17	13 or 14 or 15 or 16
18	randomized controlled trial. pt
19	controlled clinical trial. pt
20	randomized controlled trials. Mesh.
21	random allocation. Mesh.
22	randomized. ti, ab
23	randomly. ti, ab
24	double-blind method. Mesh
25	single-blind method. Mesh
26	clinical trial. pt
27	18 or 19-26
28	(3 or 6 or 9 or 12) and 17 and 27

### 2.4 Data collection and analysis

#### 2.4.1 Selection of studies

**Figure 1:**
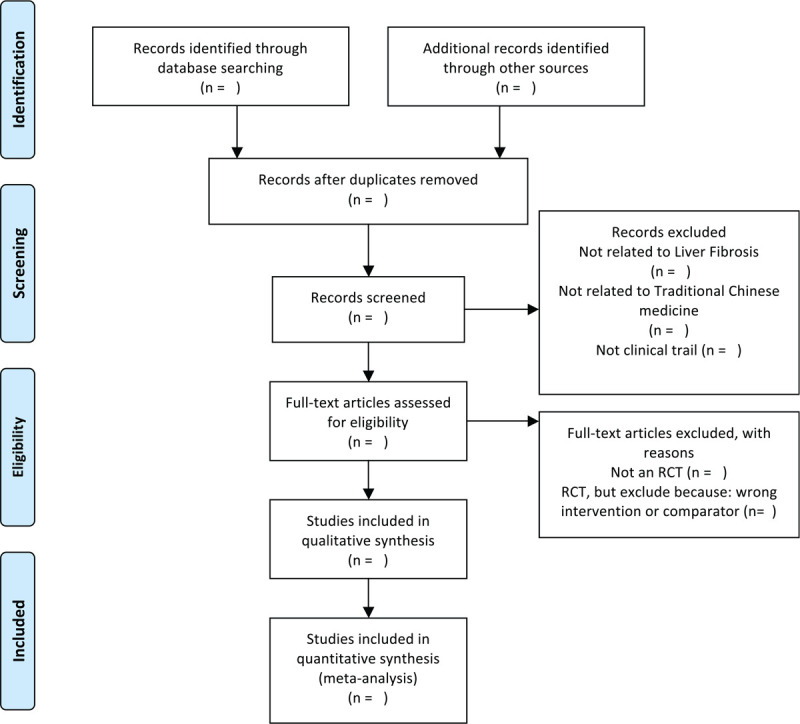
The PRISMA-P flow chart of the study selection process. PRISMA-P = the preferred reporting items for systematic reviews and meta-analyses protocols.

The titles and abstracts of all searched studies will be reviewed and screened independently by 2 reviewers, aiming at identifying eligible trials and eliminating duplicated or irrelevant studies in line with the criteria; the full text of all possibly eligible studies will obtained if necessary. A discussion with the third reviewer is planned to solve the disagreements. A PRISMA-P flow diagram will be used to show the study selection process (Fig. 1).

### 2.5 Data extraction and management

The following data will be extracted from the selected studies by 2 independent reviewers using a standard data extraction sheet: year of publication, country, general information, participant characteristics, inclusion and exclusion criteria, sample size, randomization, blinding methods, methods, type of acupuncture interventions, control, outcome measures, results, adverse reactions, conflicts of interest, ethical approval, and other information. The authors will be contacted for further information if the reported data is insufficient and the third reviewer will be set to solve the disagreements.

### 2.6 Assessment of risk of bias and reporting of study quality

Two independent reviewers will access the quality of included literature and complete the standards for reporting interventions in clinical trials of acupuncture checklist with the Cochrane collaboration risk-of-bias assessment method.^[7]^

### 2.7 Measures of treatment effect

Dichotomous data will be presented as risk ratio and 95% confidence intervals, while continuous outcomes will be shown as standard mean difference 95% confidence intervals.

### 2.8 Unit of analysis issues

The individual participant will the analytical unit.

### 2.9 Management of missing data

The cause of the missing data will be determined to solve the problem. And if this is not working, the authors will be contacted for the missing part. This will be documented and the available data will be extracted and analyzed if the missing data cannot be obtained.

### 2.10 Assessment of heterogeneity

*I*^2^ test will be used to quantify inconsistency and standard *χ*^2^ test will be used to detect statistical heterogeneity. Studies will be considered to have homogeneity if the *P*-value exceeds 0.1 and the *I*^2^ value is less than 50%, and the fixed-effects model will be used. While studies will be considered to have significant statistic heterogeneity if the *P*-value is less than .1 or the *I*^2^ value exceeds 50%, and subgroup analysis will be used to explore the possible cause. And the random-effects model will be applied if the heterogeneity is still important.

### 2.11 Assessment of reporting biases

Funnel plots will be used to assess the reporting biases if more than 10 studies are included.

### 2.12 Data synthesis

Review Manager Software V.53 will be used for data synthesis. The level of statistical heterogeneity will determine how the data will be synthesized and analyzed. The random-effects model will be used if the *I*^2^ value is no less than 50%. The fixed-effects model will be used if the heterogeneity tests show little statistical heterogeneity. If there is meaningful heterogeneity that cannot be explained by any assessment, meta-analysis will not be performed. If necessary, each subgroup will be carefully considered for subgroup analysis.

### 2.13 Subgroup analysis

Subgroup analysis will be conducted if the data are sufficient, according to the factors different outcomes and different control interventions.

### 2.14 Sensitivity analysis

Sensitivity analysis will be conducted to test the robustness of the review conclusions if possible. The impacts of sample size, study design, methodological quality, and missing data will be evaluated.

### 2.15 Grading the quality of evidence

The grade of recommendations assessment, development, and evaluation will be the tool to evaluate the quality of the evidence.^[8]^ Limitation of study design, inconsistency of results, indirectness, imprecision, and publication bias will be assessed. The assessments will be divided into 4 levels: very low, low, moderate, or high.

### 2.16 Ethics and dissemination

This protocol will not evaluate individual patient information or affect patient rights and therefore does not require ethical approval. Results from this review will be disseminated through peer-reviewed journals and conference reports.

## 3 Discussion

This systematic review will assess the effectiveness and safety of traditional Chinese medicine on liver fibrosis. There are 4 sections in the review: identification, study inclusion, data extraction, and data synthesis. This review will help the doctors to choose traditional Chinese medicine as an alternative treatment for liver fibrosis, and offer the patients more options to relieve their symptoms.

## Author contributions

**Data curation:** Xia Gao.

**Funding acquisition:** Wenfu Cao.

**Investigation:** Wenfu Cao.

**Methodology:** Wenfu Cao.

**Resources:** Wenfu Cao.

**Software:** Xia Gao.

**Supervision:** Wenfu Cao.

**Writing – original draft:** Xia Gao.

**Writing – review & editing:** Xia Gao.
